# Genetic diversity and differentiation of populations of *Chlorops oryzae* (Diptera, Chloropidae)

**DOI:** 10.1186/s12898-020-00293-8

**Published:** 2020-04-15

**Authors:** Ailin Zhou, Ping Tian, Zhongcai Li, Xinwen Li, Xiaoping Tan, Zhengbing Zhang, Lin Qiu, Hualiang He, Wenbing Ding, Youzhi Li

**Affiliations:** 1 Hunan Provincial Key Laboratory for Biology and Control of Plant Diseases and Insect Pests, College of Plant Protection, Hunan Agricultural University, Changsha, 410128 China; 2 Hunan Provincial Engineering & Technology Research Center for Biopesticide and Formulation Processing, Changsha, 410128 China; 3 Plant Protection and Inspection Station, Agriculture Bureau of Hanshou County, Hanshou, 415900 China; 4 Agriculture and Rural Department of Hunan Province, Plant Protection and Inspection Station, Changsha, 410005 China

**Keywords:** *Chlorops oryzae*, Mitochondrial DNA, ISSR, Genetic diversity, Genetic differentiation

## Abstract

**Background:**

*Chlorops oryzae* is an important pest of rice crops. There have been frequent outbreaks of this pest in recent years and it has become the main rice pest in some regions. To elucidate the molecular mechanism of frequent *C. oryzae* outbreaks, we estimated the genetic diversity and genetic differentiation of 20 geographical populations based on a dataset of ISSR markers and *COI* sequences.

**Results:**

ISSR data revealed a high level of genetic diversity among the 20 populations as measured by Shannon’s information index (*I*), Nei’s gene diversity (*H*), and the percentage of polymorphic bands (PPB). The mean coefficient of gene differentiation (*Gst*) was 0.0997, which indicates that only 9.97% genetic variation is between populations. The estimated gene flow (*Nm*) value was 4.5165, indicating a high level of gene flow and low, or medium, genetic differentiation among some populations. The results of a Mantel test revealed no significant correlation between genetic and geographic distance among populations, which means there is no evidence of significant genetic isolation by distance. An UPGMA (unweighted pair-group method with arithmetic averages) dendrogram based on genetic identity, did not indicate any major geographic structure for the 20 populations examined. mtDNA *COI* data indicates low nucleotide (0.0007) and haplotype diversity (0.36) in all populations. *Fst* values suggest that the 20 populations have low, or medium, levels of genetic differentiation. And the topology of a Neighbor-Joining tree suggests that there are no independent groups among the populations examined.

**Conclusions:**

Our results suggest that *C. oryzae* populations have high genetic diversity at the species level. There is evidence of frequent gene flow and low, or medium, levels of genetic differentiation among some populations. There is no significant correlation between genetic and geographic distance among *C. oryzae* populations, and therefore no significant isolation by distance. All results are consistent with frequent gene exchange between populations, which could increase the genetic diversity, and hence, adaptability of *C. oryzae*, thereby promoting frequent outbreaks of this pest. Such knowledge may provide a scientific basis for predicting future outbreaks.

## Background

*Chlorops oryzae* (Diptera, Chloropidae) is an important pest of paddy rice plants, which inflicts significant economic damage to rice crops throughout Asia. However, the dispersal ability, potential for long-distance dispersal, pattern of migration, ecological amplitude, and population size of *C. oryzae* are still uncertain. Most research on this species has focused on the physiology and ecology [1–3], and its genetics is relatively unstudied [4].

Frequent *C. oryzae* outbreaks in recent years have caused the species to become a major pest in some regions. The propensity for outbreaks may itself play an important role in homogenizing genetic variation and intensifying gene flow between pest populations [5, 6]. We hypothesized that frequent gene flow between populations enhances the species’ overall adaptability, promoting the frequent outbreaks that occur today. In other words, the frequent outbreaks of *C. oryzae* are associated with the species’ genetic diversity, population demography and high rate of gene flow between populations.

To test this hypothesis, we evaluated the genetic structure of different geographic populations of *C. oryzae* and the level of gene flow between them. We quantified the genetic diversity and degrees of genetic differentiation of 20 different geographical populations, which may provide a scientific basis for predicting future outbreaks.

We used two effective and promising DNA markers, mitochondrial DNA (mtDNA) and inter-simple sequence repeat (ISSR), to examine between-population differences. Studies of genetic variation between pest populations can not only provide information on their population structure in different geographical regions, but also deduce the demographic history of this species [7–9]. Yi et al. used microsatellite and mtDNA loci to investigate the genetic divergence and dispersal ability of *Bactrocera dorsalis* (Hendel) on six offshore islands in South China, which results indicated that these populations have high genetic diversity, frequent gene flow and low, or medium, levels of genetic differentiation. Thus, the geographic isolation of the six islands is no barrier to the dispersal of *B. dorsalis* [10]. Research on the genetic diversity and population structure of *Leucinodes orbonalis* collected from a variety of agro-climatic conditions found almost no genetic diversity and no significant genetic variation among the mitochondrial gene Cytochrome Oxidase I (*COI*) gene sequences of the populations examined. However, a few genetically distinct populations were associated with some specific habitat requirements [11]. Similarly, genetic differentiation in ISSR markers and the *COI* gene among Iranian populations of *Hishimonus phycitis* may have been induced by geographical and ecological isolation and may have an impact on the vectoring capability of this insect [12].

Our results not only provide information on the genetic structure and phylogeography research of *C. oryzae*, but also provide a potential scientific basis for monitoring and controlling this pest.

## Results

### *COI* gene analysis

#### Genetic diversity and differentiation

In total, 432 individuals collected from different locations were used to amplify 684 bp of the *COI* gene sequence, which defined 47 haplotypes. All 47 haplotypes had 43 variable sites, including 26 singleton variable sites and 17 parsimony informative sites. The mean total nucleotide frequencies of A, T, C and G in the nucleotide sequences from the 20 different populations were 29.98%, 36.56%, 16.95% and 16.51%, respectively, which shows an obvious AT bias (66.54%). The transition/transversion rate ratio was observed to be higher with purines (36.158) than pyrimidines (19.381). The overall transition/transversion bias was 12.022.

Genetic diversity parameters of the 20 populations and the results of neutrality tests are shown in Table 1. Haplotype diversity (*Hd*) for each population ranged from 0 to 0.71739 and the average number of differences (*k*) ranged from 0 to 1.35145. Nucleotide diversity (*Π*) for each population ranged from 0 to 0.00198. Tajima’s *D* and Fu’s *Fs* test of neutrality of 19 populations showed a negative value. When all samples were calculated as one population, Tajima’s *D* and Fu’s *Fs* values were negative and 1‰ significant, which is strong evidence of population expansion.

**Table 1:** Genetic diversity of 20 *C. oryzae* populations

populations	n	h	Hd	K	Pi	Tajima’s D	P-value	Fu’s Fs	P-value
TY	24	4	0.23913	0.25000	0.00037	− 1.73253	0.10 > P > 0.05	− 3.021	0.039
ZZ	24	3	0.16304	0.16667	0.00024	− 1.51469	> 0.10	− 2.078	0.094
LS	24	4	0.23913	0.25000	0.00037	− 1.73253	0.10. > P > 0.05	− 3.021	0.039
YS	24	3	0.16304	0.25000	0.00037	− 1.73253	0.10 > P > 0.05	− 1.355	0.159
XT	24	3	0.23551	0.24275	0.00035	− 1.20229	> 0.10	− 1.407	0.153
HS	24	3	0.23551	0.24275	0.00035	− 1.20229	> 0.10	− 1.407	0.153
HD	16	5	0.60833	1.07500	0.00157	− 1.38795	> 0.10	− 1.243	0.145
JS	16	4	0.35000	0.37500	0.00055	− 1.69654	0.10 > P > 0.05	− 2.449	0.065
SM	24	5	0.31159	0.33333	0.00049	− 1.88381	< 0.05	− 3.974	0.016
DC	24	6	0.49638	0.64493	0.00094	− 1.81040	< 0.05	− 3.383	0.026
XX	24	8	0.56159	0.65942	0.00096	− 2.02600	< 0.05	− 6.535	0.001
YL	8	1	0	0	0	N	N	N	N
YX	24	3	0.30072	0.31159	0.00046	− 0.91964	> 0.10	− 0.960	0.200
SS	24	4	0.23913	0.33333	0.00049	− 1.88381	< 0.05	− 2.331	0.070
LL	24	7	0.71739	1.15942	0.00170	− 1.19233	> 0.10	− 2.620	0.047
TJ	24	4	0.23913	0.25000	0.00037	− 1.73253	0.10 > P> 0.05	− 3.021	0.039
LH	24	6	0.38043	0.41667	0.00061	− 1.99611	< 0.05	− 4.976	0.006
GZ	8	2	0.25000	0.25000	0.00037	− 1.05482	> 0.10	− 0.182	0.354
ZJ	24	5	0.37681	0.48551	0.00071	− 1.49528	> 0.10	− 2.842	0.043
NX	24	9	0.66304	1.35145	0.00198	− 1.98791	< 0.05	− 4.496	0.008
Total	432	47	0.36000	0.48000	0.00070	− 2.56478	< 0.001	− 100.982	0.000

n: Number of individual, h: Number of haplotype, Hd: Haplotype diversity, K: Average number of differences, Pi: Nucleotide diversity

Based on our sequence database, inter- and intraspecific genetic distances of *C. oryzae* populations were 0.00012–0.00184 and 0–0.00198 (Table 2) respectively, which indicates no significant genetic differentiation. The *Fst* values between populations ranged from − 0.0595 to 0.1174 (Table 3), indicating that inter-population differences are relatively low. AMOVA results suggest that 97.28% of all genetic variation is within, and only 2.72% between, populations (Table 4).

**Table 2:** Genetic distances between (below diagonal) and within *C. oryzae* populations (on diagonal) based on *COI* sequences

	TY	ZZ	LS	YS	XT	HS	HD	JS	SM	DC	XX	YL	YX	SS	LL	TJ	LH	GZ	ZJ	NX
TY	0.00037																			
ZZ	0.00031	0.00024																		
LS	0.00037	0.00031	0.00037																	
YS	0.00037	0.00031	0.00036	0.00037																
XT	0.00037	0.00030	0.00037	0.00037	0.00036															
HS	0.00037	0.00031	0.00036	0.00036	0.00037	0.00036														
HD	0.00101	0.00093	0.00101	0.00101	0.00098	0.00101	0.00158													
JS	0.00046	0.00040	0.00046	0.00046	0.00046	0.00046	0.00110	0.00055												
SM	0.00043	0.00037	0.00043	0.00043	0.00043	0.00043	0.00105	0.00052	0.00049											
DC	0.00067	0.00059	0.00067	0.00067	0.00064	0.00067	0.00127	0.00076	0.00073	0.00095										
XX	0.00067	0.00060	0.00067	0.00067	0.00065	0.00067	0.00127	0.00076	0.00073	0.00094	0.00097									
YL	0.00018	0.00012	0.00018	0.00018	0.00018	0.00018	0.00083	0.00028	0.00024	0.00049	0.00048	0.00000								
YX	0.00042	0.00035	0.00043	0.00043	0.00040	0.00043	0.00102	0.00052	0.00049	0.00068	0.00070	0.00024	0.00046							
SS	0.00043	0.00037	0.00043	0.00043	0.00043	0.00043	0.00105	0.00052	0.00048	0.00073	0.00073	0.00024	0.00049	0.00049						
LL	0.00116	0.00109	0.00116	0.00116	0.00116	0.00116	0.00167	0.00125	0.00119	0.00146	0.00142	0.00098	0.00122	0.00119	0.00170					
TJ	0.00037	0.00030	0.00037	0.00037	0.00037	0.00037	0.00098	0.00046	0.00042	0.00065	0.00066	0.00018	0.00041	0.00042	0.00113	0.00037				
LH	0.00049	0.00043	0.00049	0.00049	0.00049	0.00048	0.00113	0.00058	0.00055	0.00079	0.00079	0.00031	0.00055	0.00055	0.00128	0.00049	0.00061			
GZ	0.00037	0.00031	0.00035	0.00035	0.00035	0.00035	0.00101	0.00046	0.00043	0.00066	0.00067	0.00018	0.00043	0.00043	0.00116	0.00037	0.00049	0.00037		
ZJ	0.00055	0.00049	0.00055	0.00055	0.00055	0.00055	0.00117	0.00064	0.00061	0.00086	0.00085	0.00037	0.00061	0.00061	0.00122	0.00055	0.00067	0.00055	0.00071	
NX	0.00122	0.00116	0.00122	0.00122	0.00121	0.00122	0.00177	0.00131	0.00126	0.00151	0.00148	0.00104	0.00127	0.00126	0.00184	0.00120	0.00134	0.00122	0.00140	0.00198

**Table 3:** Genetic differentiation (pairwise *F*_*ST*_) of 20 *C. oryzae* populations based on nucleotide sequences of mitochondrial DNA. Lower left diagonal represents the

	TY	ZZ	LS	YS	XT	HS	HD	JS	SM	DC	XX	YL	YX	SS	LL	TJ	LH	GZ	NX	ZJ
TY		–	–	–	–	–	+	–	–	–	–	–	–	–	+	–	–	–	+	–
ZZ	0.0000		–	–	–	–	–	–	–	–	–	–	–	–	+	–	–	–	+	–
LS	0.0000	0.0000		–	–	–	+	–	–	–	–	–	–	–	+	–	–	–	+	–
YS	0.0000	0.0000	− 0.0141		–	–	+	–	–	–	–	–	–	–	+	–	–	–	+	–
XT	0.0145	− 0.0165	0.0145	0.0145		–	–	–	–	–	–	–	–	–	+	–	–	–	–	–
HS	0.0145	0.0174	0.0006	0.0006	0.0290		+	–	–	–	–	–	–	–	+	–	–	–	+	–
HD	0.0555	0.0442	0.0555	0.0555	0.0284	0.0616		–	–	–	–	–	–	–	–	–	+	–	–	–
JS	0.0044	0.0089	0.0044	0.0044	0.0168	0.0168	0.0333		–	–	–	–	–	–	+	–	–	–	–	–
SM	0.0000	0.0000	0.0000	0.0000	0.0124	0.0124	0.0347	0.0013		–	–	–	–	–	+	–	–	–	–	–
DC	0.0163	− 0.0075	0.0163	0.0163	− 0.0145	0.0242	0.0112	0.0146	0.0217		–	–	–	–	+	–	–	–	+	–
XX	0.0003	− 0.0167	0.0079	0.0079	− 0.0150	0.0158	0.0022	0.0011	0.0003	− 0.0153		–	–	–	+	–	–	–	–	–
YL	− 0.0595	− 0.0595	− 0.0595	− 0.0595	− 0.0381	− 0.0381	− 0.0140	− 0.0503	− 0.0595	− 0.0354	− 0.0515		–	–	–	–	–	–	–	–
YX	0.0257	0.0019	0.0373	0.0373	− 0.0234	0.0497	0.0204	0.0332	0.0326	− 0.0279	− 0.0132	− 0.0108			+	–	–	–	+	+
SS	0.0000	0.0000	0.0000	0.0000	0.0124	0.0124	0.0347	0.0013	− 0.0105	0.0217	0.0003	− 0.0595	0.0326		+	–	–	–	–	–
LL	0.1098	0.1118	0.1098	0.1098	0.1144	0.1144	0.0151	0.0846	0.0774	0.0947	0.0612	0.0400	0.1174	0.0774		+	+	–	–	+
TJ	0.0000	− 0.0170	0.0000	0.0000	− 0.0137	0.0145	0.0221	0.0044	− 0.0121	− 0.0068	− 0.0152	− 0.0595	0.0016	− 0.0121	0.0816		–	–	–	–
LH	0.0000	0.0000	0.0000	0.0000	0.0109	− 0.0102	0.0452	− 0.0011	0.0000	0.0201	0.0067	− 0.0595	0.0290	0.0000	0.0994	0.0000		–	+	–
GZ	0.0000	0.0189	− 0.0435	− 0.0435	0.0158	− 0.0272	− 0.0024	− 0.0123	− 0.0116	− 0.0309	− 0.0255	0.0000	0.0269	− 0.0116	0.0480	0.0000	− 0.0194		–	–
NX	0.0351	0.0370	0.0391	0.0391	0.0354	0.0435	− 0.0064	0.0224	0.0217	0.0288	0.0066	− 0.0253	0.0382	0.0217	0.0012	0.0187	0.0356	− 0.0137		+
ZJ	0.0193	0.0217	0.0193	0.0193	0.0290	0.0290	0.0309	0.0135	0.0174	0.0311	0.0186	− 0.0381	0.0435	0.0174	0.0890	0.0193	0.0158	− 0.0068	0.0346	

*F*_*ST*_ value and upper diagonal showing the significance (+, P < 0.05)

**Table 4:** AMOVA analysis of mtDNA *COI* gene sequences in 20 *C. oryzae* populations

Source of variation	df	Sum of squares	Variance components	Percentage of variation
Among populations	19	7.116	0.00654Va	2.72
Within populations	412	96.250	0.23362Vb	97.28
Total	431	103.366	0.24016	100

*Fst *= 0.02724 (*P* = 0.000)

#### Haplotype network and population tree

**Fig. 1:**
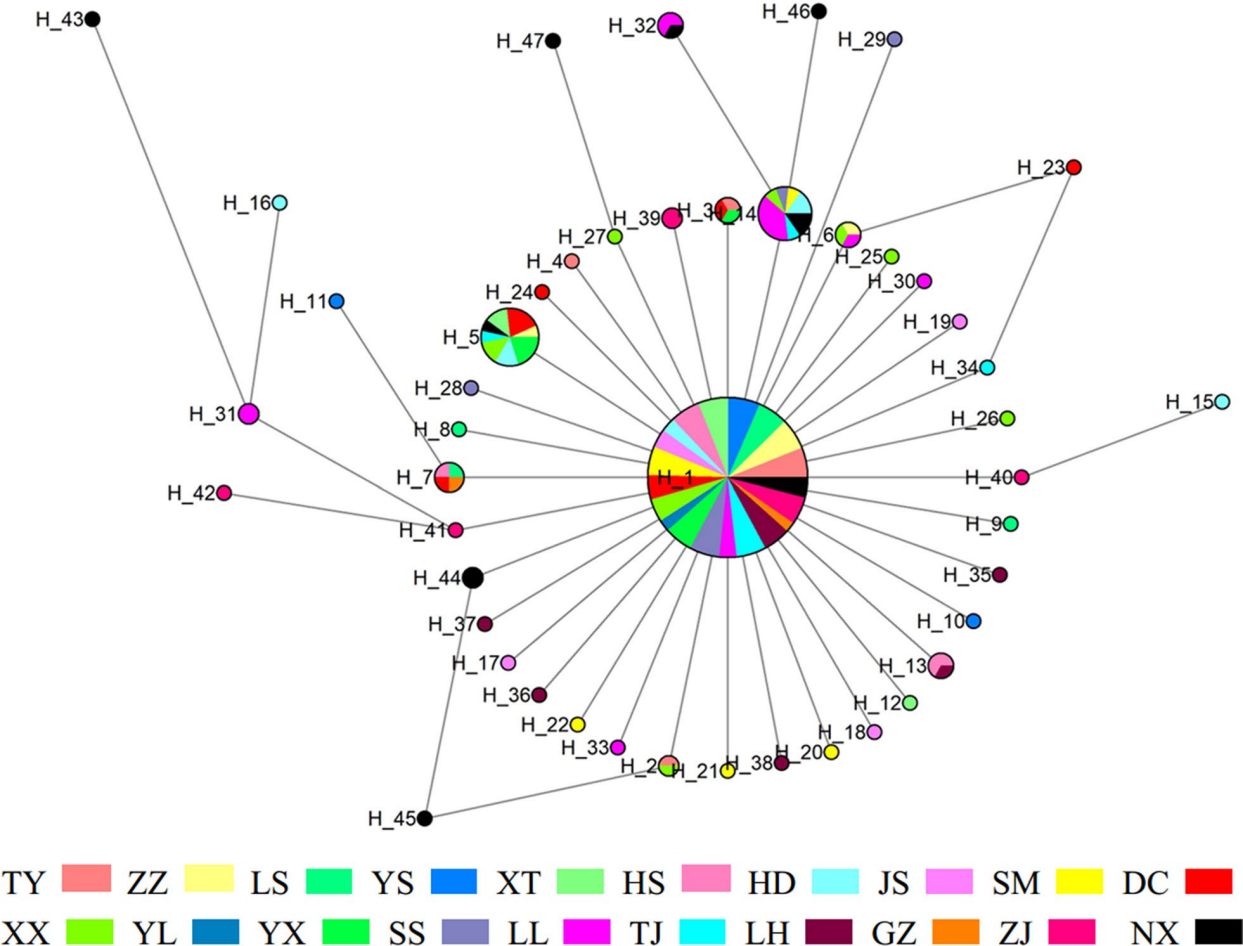
Median-joining network based on the single gene of *COI* haplotypes. Each circle represents a haplotype, and the area of a circle is proportional to the number of individuals with that haplotype. Colors within nodes refer to *C. oryzae* sampling regions

**Fig. 2:**
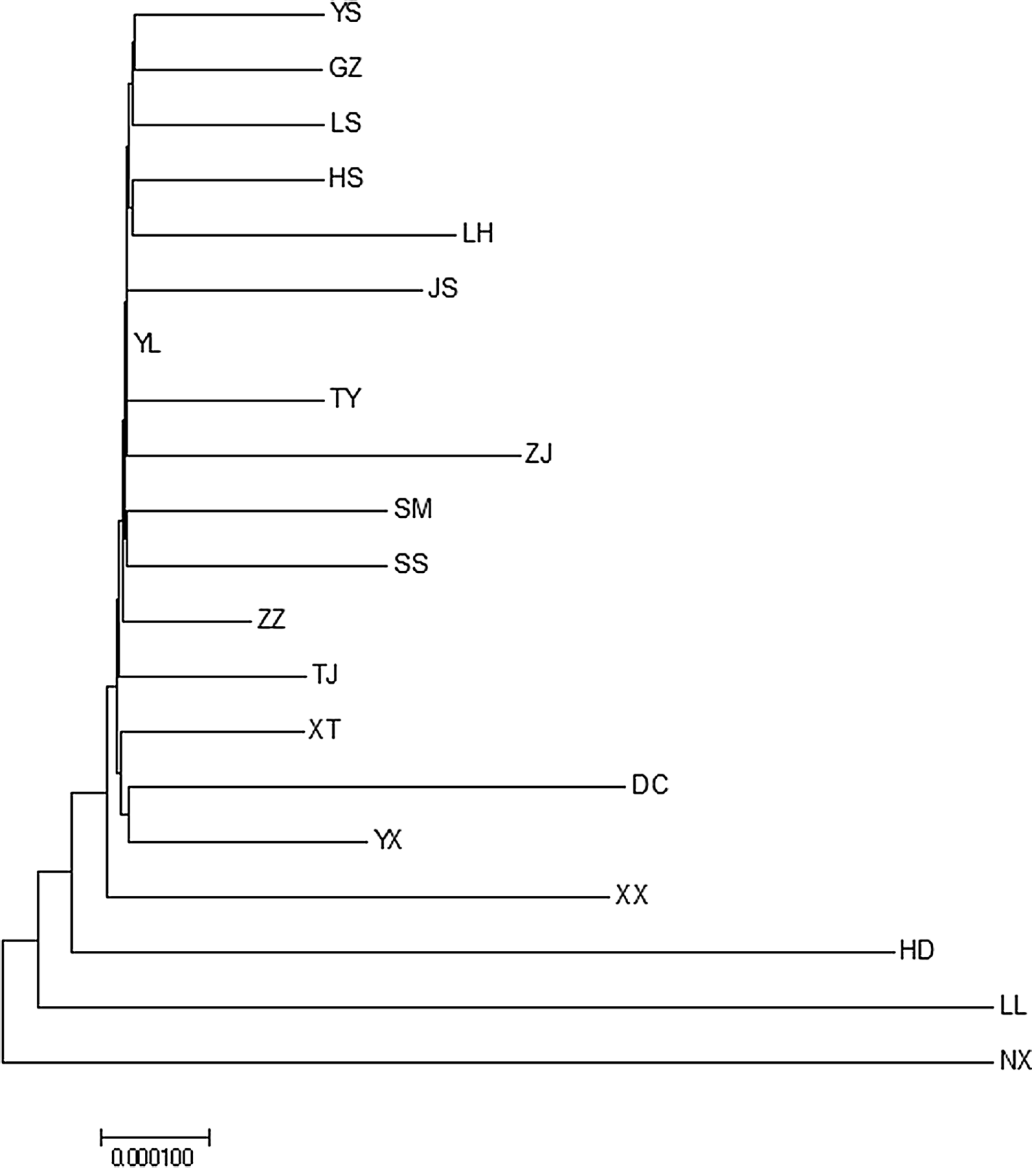
Neighbor-joining tree (model Kimura-2 parameter) of the phylogenetic relationships among 20 *C. oryzae* populations based on *COI* gene variation (1000 bootstrap replicates)

Evolutionary relationships among the haplotypes were depicted using the median-joining network method (Fig. 1). Among the 47 haplotypes, H1 was the most frequent haplotype, which occupied a central position of the network and was diversified by 46 haplotypes. Especially, H23 and H45 can be derived from two different haplotypes with just one mutational step, respectively. The topology of the *C. oryzae* population Neighbor-Joining tree suggested that there were no independent groups in all populations (Fig. 2). It is worth mentioning that NX population is most distant on the haplotype network and population tree, however, there is no certain landscape features around the NX location. The reason for preventing emigration of individuals is still unknown.

### ISSR-PCR analysis

#### Genetic diversity

197 fragments were generated from the 9 primers, ranging in size from 250 to 2000 bp, and with an average of 21.89 fragments per primer. The percentage of polymorphic bands was 100% showing high genetic diversity at the species level. Among the 20 populations, PPB values ranged from 89.85% to 99.49%. The *Na* and *Ne* were 2.0000 ± 0.0000 (1.8985 ± 0.3028 to 1.9949 ± 0.0712 among populations) and 1.6460 ± 0.2373 (1.4992 ± 0.3270 to 1.6908 ± 0.2784 among populations), respectively. The *H* and *I* were 0.3785 ± 0.0984 (0.2992 ± 0.1588 to 0.3893 ± 0.1196) and 0.5609 ± 0.1151 (0.4547 ± 0.2091 to 0.5692 ± 0.1457), respectively, within all samples (Table 5).

**Table 5:** Genetic variation among *C. oryzae* populations

Population	Number of polymorphic bands	PPB (%)	*Na*	*Ne*	*H*	*I*
TY	189	95.94	1.9594 ± 0.1979	1.5564 ± 0.2981	0.3307 ± 0.1428	0.4970 ± 0.1848
ZZ	194	98.48	1.9848 ± 0.1228	1.5755 ± 0.2915	0.3410 ± 0.1342	0.5119 ± 0.1683
LS	192	97.46	1.9746 ± 0.1577	1.6440 ± 0.2820	0.3704 ± 0.1275	0.5460 ± 0.1612
YS	190	96.45	1.9645 ± 0.1856	1.5740 ± 0.2886	0.3404 ± 0.1355	0.5101 ± 0.1736
XT	189	95.94	1.9594 ± 0.1979	1.6300 ± 0.3049	0.3604 ± 0.1431	0.5309 ± 0.1855
HS	194	98.48	1.9848 ± 0.1228	1.6908 ± 0.2784	0.3893 ± 0.1196	0.5692 ± 0.1457
HD	191	96.95	1.9695 ± 0.1723	1.5777 ± 0.2993	0.3403 ± 0.1395	0.5095 ± 0.1773
JS	187	94.92	1.9492 ± 0.2201	1.5680 ± 0.3006	0.3360 ± 0.1403	0.5036 ± 0.1817
SM	194	98.48	1.9848 ± 0.1228	1.6039 ± 0.2831	0.3545 ± 0.1278	0.5287 ± 0.1584
DC	196	99.49	1.9949 ± 0.0712	1.5609 ± 0.2835	0.3367 ± 0.1272	0.5091 ± 0.1556
XX	195	98.98	1.9898 ± 0.1005	1.5200 ± 0.2769	0.3193 ± 0.1283	0.4881 ± 0.1596
YL	177	89.85	1.8985 ± 0.3028	1.5646 ± 0.3245	0.3289 ± 0.1578	0.4893 ± 0.2113
YX	192	97.46	1.9746 ± 0.1577	1.5377 ± 0.2914	0.3243 ± 0.1370	0.4916 ± 0.1737
SS	187	94.92	1.9492 ± 0.2201	1.5917 ± 0.3165	0.3426 ± 0.1501	0.5087 ± 0.1955
LL	192	97.46	1.9746 ± 0.1577	1.6025 ± 0.2834	0.3539 ± 0.1281	0.5275 ± 0.1615
TJ	195	98.98	1.9898 ± 0.1005	1.5816 ± 0.2890	0.3443 ± 0.1310	0.5167 ± 0.1621
LH	195	98.98	1.9898 ± 0.1005	1.5947 ± 0.2691	0.3536 ± 0.1172	0.5300 ± 0.1420
GZ	180	91.37	1.9137 ± 0.2815	1.5762 ± 0.3268	0.3342 ± 0.1551	0.4969 ± 0.2048
ZJ	185	93.91	1.9391 ± 0.2398	1.4992 ± 0.3270	0.2992 ± 0.1588	0.4547 ± 0.2091
NX	190	96.45	1.9645 ± 0.1856	1.5801 ± 0.2966	0.3416 ± 0.1393	0.5106 ± 0.1791
Total	197	100.00	2.0000 ± 0.0000	1.6460 ± 0.2373	0.3785 ± 0.0984	0.5609 ± 0.1151

*PPB* the percentage of polymorphic bands, *Na* Observed number of alleles, *Ne* effective number of alleles

*H* = Nei’s (1973) gene diversity, *I* = Shannon’s information index

#### Genetic differentiation

The *Gst* was 0.0997, which indicates that 90.03% genetic variation is within populations and only 9.97% between populations. The estimated *Nm* value was 4.5165 (Table 6). These results suggest that genetic differentiation among populations of *C. oryzae* is impeded by high gene flow. Table 7 lists the genetic identity (above diagonal) and genetic distance (below diagonal) among populations.

**Table 6:** Population genetic differentiation coefficients and gene flow among *C. oryzae* populations

	Total genetic diversity (*Ht*)	Genetic diversity within populations (*Hs*)	Coefficient of gene differentiation (*Gst*)	Gene flow (*Nm*)
Mean	0.3799	0.3421	0.0997	4.5165
Standard deviation	0.0095	0.0074

**Table 7:** Nei’s genetic identity (above diagonal) and genetic distance (below diagonal) among *C. oryzae* populations

	TY	ZZ	LS	YS	XT	HS	HD	JS	SM	DC	XX	YL	YX	SS	LL	TJ	LH	GZ	ZJ	NX
TY		0.9644	0.9643	0.9690	0.9543	0.9680	0.9770	0.9630	0.9614	0.9665	0.9638	0.9545	0.9533	0.9583	0.9633	0.9630	0.9521	0.9216	0.9473	0.9616
ZZ	0.0362		0.9657	0.9800	0.9430	0.9547	0.9627	0.9494	0.9494	0.9533	0.9510	0.9342	0.9439	0.9406	0.9632	0.9580	0.9456	0.9025	0.9157	0.9388
LS	0.0363	0.0349		0.9652	0.9694	0.9643	0.9698	0.9606	0.9566	0.9505	0.9624	0.9444	0.9568	0.9556	0.9680	0.9553	0.9558	0.9259	0.9209	0.9589
YS	0.0315	0.0202	0.0354		0.9493	0.9560	0.9712	0.9555	0.9508	0.9593	0.9592	0.9362	0.9523	0.9428	0.9676	0.9604	0.9460	0.9131	0.9273	0.9415
XT	0.0468	0.0586	0.0311	0.0520		0.9770	0.9643	0.9565	0.9475	0.9472	0.9620	0.9477	0.9512	0.9593	0.9599	0.9406	0.9457	0.9198	0.9239	0.9516
HS	0.0325	0.0463	0.0363	0.0450	0.0232		0.9730	0.9582	0.9607	0.9636	0.9544	0.9572	0.9487	0.9721	0.9684	0.9514	0.9567	0.9406	0.9357	0.9628
HD	0.0233	0.0380	0.0306	0.0292	0.0364	0.0274		0.9778	0.9599	0.9619	0.9695	0.9568	0.9617	0.9710	0.9714	0.9609	0.9585	0.9261	0.9420	0.9608
JS	0.0377	0.0519	0.0402	0.0455	0.0444	0.0427	0.0225		0.9481	0.9632	0.9790	0.9569	0.9738	0.9554	0.9705	0.9563	0.9672	0.9221	0.9407	0.9630
SM	0.0394	0.0519	0.0444	0.0505	0.0539	0.0401	0.0409	0.0533		0.9553	0.9580	0.9390	0.9453	0.9489	0.9599	0.9594	0.9599	0.9398	0.9426	0.9728
DC	0.0341	0.0479	0.0508	0.0415	0.0543	0.0371	0.0389	0.0375	0.0457		0.9678	0.9350	0.9681	0.9446	0.9773	0.9778	0.9765	0.9332	0.9301	0.9600
XX	0.0368	0.0502	0.0384	0.0416	0.0387	0.0467	0.0309	0.0212	0.0429	0.0327		0.9497	0.9816	0.9541	0.9726	0.9588	0.9666	0.9258	0.9361	0.9616
YL	0.0466	0.0681	0.0572	0.0659	0.0537	0.0437	0.0441	0.0441	0.0629	0.0672	0.0516		0.9446	0.9532	0.9433	0.9263	0.9312	0.9090	0.9350	0.9385
YX	0.0479	0.0577	0.0442	0.0489	0.0500	0.0526	0.0390	0.0266	0.0563	0.0324	0.0185	0.0569		0.9460	0.9692	0.9622	0.9706	0.9279	0.9395	0.9557
SS	0.0426	0.0613	0.0454	0.0589	0.0416	0.0283	0.0295	0.0457	0.0525	0.0570	0.0470	0.0479	0.0555		0.9568	0.9435	0.9378	0.9203	0.9300	0.9487
LL	0.0374	0.0375	0.0325	0.0329	0.0409	0.0321	0.0290	0.0299	0.0409	0.0229	0.0278	0.0583	0.0313	0.0442		0.9645	0.9694	0.9349	0.9301	0.9606
TJ	0.0377	0.0429	0.0457	0.0404	0.0612	0.0498	0.0399	0.0447	0.0414	0.0224	0.0420	0.0765	0.0385	0.0582	0.0362		0.9755	0.9334	0.9322	0.9557
LH	0.0490	0.0560	0.0452	0.0555	0.0558	0.0443	0.0424	0.0333	0.0409	0.0237	0.0339	0.0713	0.0299	0.0642	0.0311	0.0248		0.9524	0.9445	0.9691
GZ	0.0816	0.1026	0.0770	0.0909	0.0835	0.0612	0.0768	0.0811	0.0621	0.0691	0.0771	0.0954	0.0749	0.0831	0.0673	0.0689	0.0487		0.9345	0.9551
ZJ	0.0541	0.0881	0.0825	0.0754	0.0792	0.0664	0.0597	0.0612	0.0591	0.0724	0.0661	0.0672	0.0624	0.0726	0.0724	0.0702	0.0571	0.0678		0.9583
NX	0.0392	0.0631	0.0419	0.0603	0.0496	0.0379	0.0400	0.0377	0.0275	0.0408	0.0392	0.0634	0.0453	0.0527	0.0402	0.0453	0.0314	0.0459	0.0426	

**Fig. 3:**
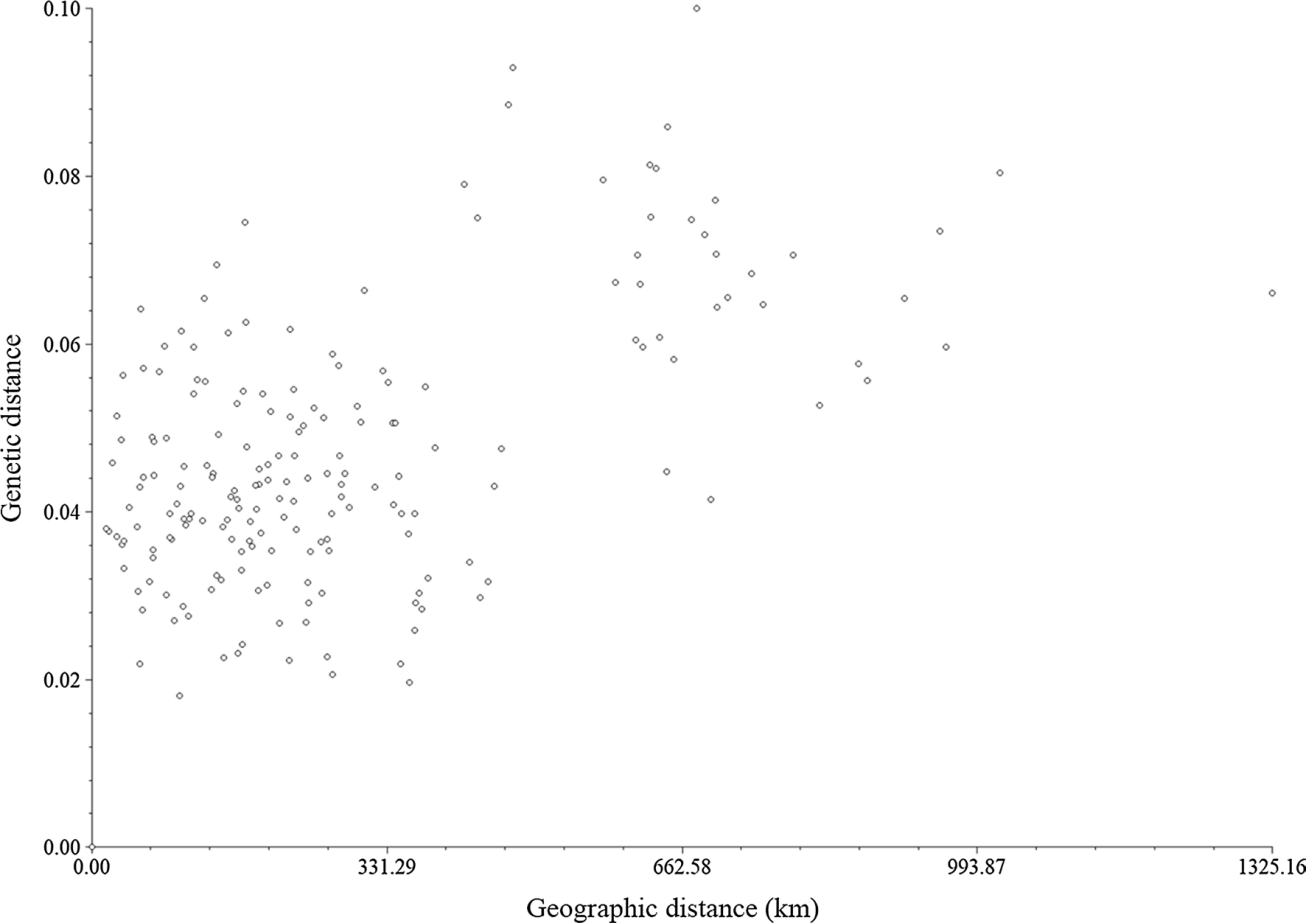
Relationship between genetic distance and geographic distance of 20 *C. oryzae* populations

The relationship between genetic and geographic distance was shown in Fig. 3. A Mantel test revealed no significant correlation between genetic and geographic distance (r = 0.54675, p = 0.9992) among *C. oryzae* populations, and there was therefore no evidence of significant isolation by distance.

**Fig. 4:**
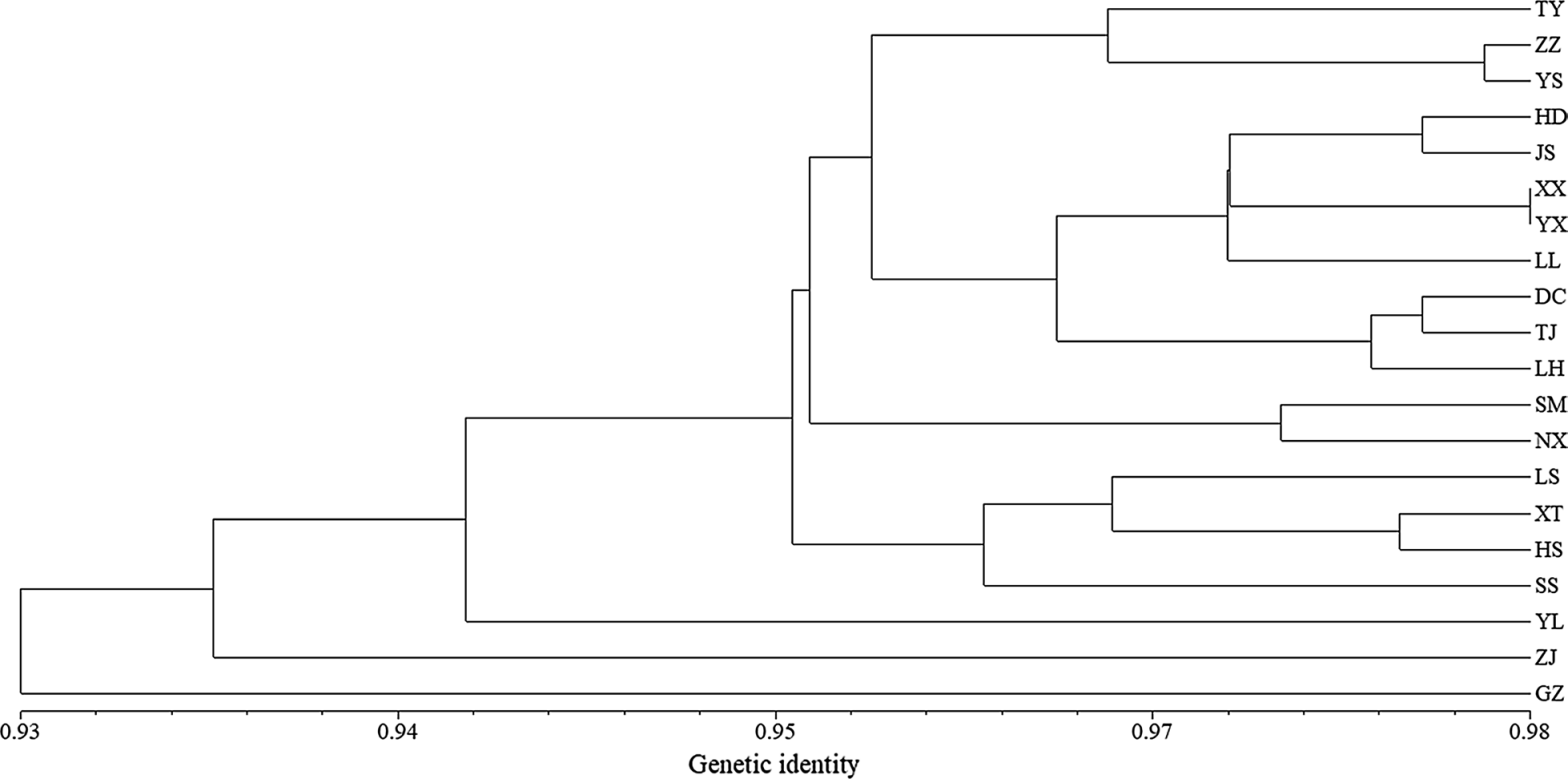
UPGMA dendrogram of 20 *C. oryzae* populations

An UPGMA dendrogram constructed based on genetic identity (Fig. 4) grouped the 20 populations into two major clusters. The dendrogram did not reveal any major geographic structure for these populations.

## Discussion

The rapid development of molecular techniques has made it possible to directly measure genetic differentiation and genetic diversity among populations [13]. The faster mutation rate and relatively conserved sequence of the mtDNA *COI* gene is ideally suited to species identification via DNA barcoding [14]. Analysis of mt*COI* gene variation is regarded as an important and reliable tool for defining cryptic species [15], evaluating biodiversity [16], identifying samples [17], and distinguishing closely related species [18].

Various surveys have demonstrated the reliability of ISSR markers, which can generate more polymorphisms than either RAPD or RFLP, [19]. For example, *Dioscorea hispida* were grouped into 10 vital groups based on information that provided by ISSR markers, proving the existence of significant variation among germplasm specimens. *D. hispida* shows a high level of genetic diversity among accessions, which suggests that ISSR markers have been very effective in detecting polymorphism in this species [20]. ISSR primers have been used to determine the potential for the diversification of cassava crops in Angola, revealing genetic diversity within populations and genetic information sharing among the three main taxa [21]. The ISSR molecular marker technique has also been used to distinguish between citrus rootstock species, and to reveal the broad genetic base and high genetic variability among these [22].

### Genetic diversity

Genetic diversity, also known as gene diversity, is the foundation of biodiversity that guarantees the evolution of species. The percentage of polymorphism, haplotype diversity and nucleotide diversity are used to evaluate population genetic diversity [10]. High levels of genetic diversity are indicative of the strong viability and adaptability of populations [23]. Results from this study indicate that the genetic diversity of *C. oryzae* was high between populations that were sampled. This may increase fitness in populations to changing conditions. Crawford and Whitney [24] also showed that genetic diversity increases the ability of species to colonize on a short-term ecological timescale by increasing the possibility of population survival, growth and reproduction under novel environments. *Episyrphus balteatus* and *Sphaerophoria scripta* European populations successfully adapt to changing environmental conditions and have great colonization abilities due to the high genetic diversity [25]. The Tajima’s *D* and Fu’s *Fs* test of neutrality values for 19 populations were negative. When all samples were calculated as one population, Tajima’s *D* and Fu’s *Fs* values were negative and 1‰ significant, strongly indicating population expansion. This result is consistent with the idea that the negative Tajima’s *D* and Fu’s *Fs* test values demonstrated demographic expansion has occurred in these populations.

### Genetic differentiation

*Fst* values between *C. oryzae* populations ranged from − 0.0595 to 0.1174, and the *Gst* value was 0.0997, both of which are indicative of low genetic differentiation between populations. This suggests frequent gene flow between populations, which may increase the species’ adaptability to environmental change [26]. Moreover, gene flow can not only demonstrate the probable genetic differentiation and genetic infiltration among populations, but also reduce the genetic differences among populations [27]. In this research, the *Nm* value of *C. oryzae* was 4.5165 which indicates high gene flow and low, or medium, genetic differentiation among some populations. High gene flow may impede genetic differentiation in *C. oryzae*. Gene flow (or lack of gene flow) plays a crucial role in genetic differentiation, affects the overall adaption of entire species and adaptative divergence between populations [28]. It has been traditionally considered as a homogeneous force that limits adaptive differences [29, 30], and recent studies have shown that it can also promote adaptation to local environmental conditions [31, 32]. For example, moderate gene flow increases the adaptation capabilities of *Rhagoletis cerasi* populations (which occupy different habitates in fragmented landscapes) to local habitates, thus preventing them from becoming extinct due to genetic processes [33, 34].

An AMOVA based on *Fst* values indicates that most of the genetic variation was resulted from the difference within populations. Furthermore, a Mantel test indicates no significant correlation between genetic and geographic distance. This result is consistent with the findings of Yang et al. [35] who compared correlation of the symmetric matrix constituted by geographic and genetic distances to analyze the existence of isolation among populations of *Odontotermes formosanus* in different regions. These authors found no significant correlation between geographic distance and genetic distance and no significant isolation by distance. Overall, we found a high level of genetic diversity and a low degree of population differentiation among populations of *C. oryzae*, and the gene flow was unaffected by geographic distance. Similarly, geographic distance did not appear to affect gene flow between 10 geographically separated populations *Oedaleus infernalis* [36]. *Fst* and *Gst* values for these populations are low, and the gene flow is high, indicating a low level of genetic differentiation and high gene flow among populations [36]. The correlation between genetic and geographic distance was insignificant [36].

Our results provide important, new information on the genetic diversity and genetic differentiation of *C. oryzae*, and suggest that high gene flow between populations contributes to the now frequent outbreaks of this pest. However, further research on both additional geographical populations and different genetic markers are necessary before definitive conclusions can be reached. Furthermore, future work can focus on doing a more comprehensive ecological and behavioural research to understand the natural history of *C. oryzae* in greater detail.

## Conclusions

This study showed that the now frequent outbreaks of *C. oryzae* may due to high gene flow between populations. We have found that these populations have high genetic diversity at the species level, whereas exhibited low genetic differentiation. High genetic diversity and frequent gene flow between populations may enhance the tolerance of populations to environmental variability and increase the adaptability to novel environmental pressures, leading to frequent outbreaks what had happened and what will happen in a large scale.

## Methods

### Sample collection and DNA extraction

**Fig. 5:**
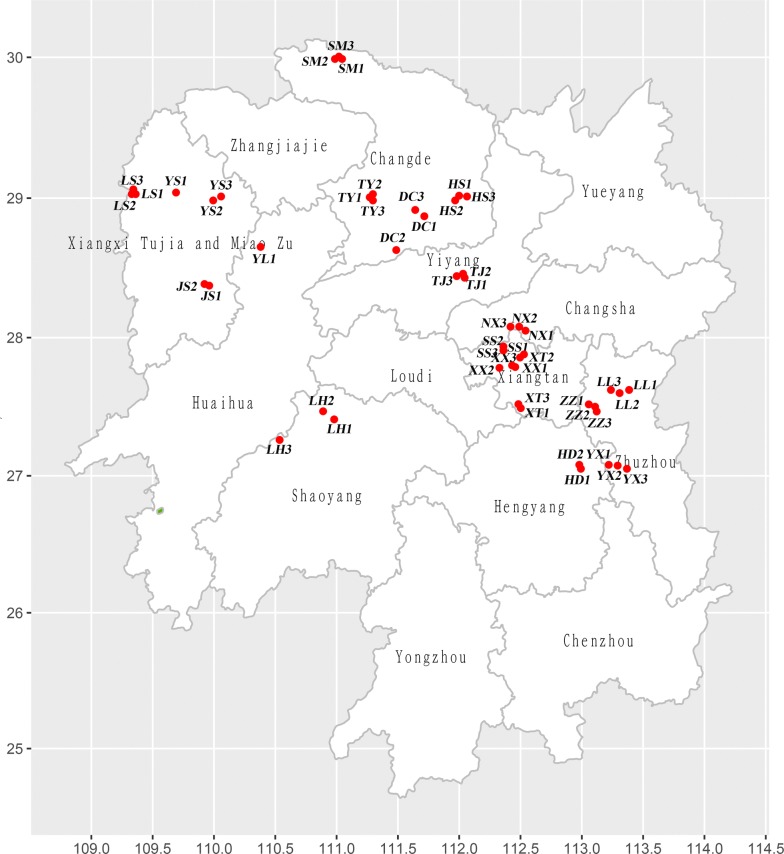
Collection sites of populations. ZJ and GZ are populations from Zhejiang and Guizhou province, about 900 km and 810 km from Hunan province, respectively. They are not shown on the figure. The figure was created by Ailin Zhou

400 specimens of *C. oryzae* were collected from different parts of Hunan province, China, and an additional 32 specimens from Zhejiang and Guizhou provinces (Fig. 5, Additional file 1: Table S1). Samples were soaked in 100% ethanol and stored at − 20 °C until their genomic DNA (gDNA) was isolated. After removing the residual ethanol, DNA was extracted from each individual using an Ezup Column Animal Genomic DNA Extraction Kit as per the manufacturer’s instructions (Sangon Biotech, Shanghai, China).

### *COI* PCR amplification and sequencing

The *COI* was amplified with the *COI*F (5′-CTAGGTGCTCCAGATATAGCATTTC-3′) and *COI*R (5′-GGCTAAAACAACTCCTGTTAATCC-3′) primers from isolated DNA. PCR was performed in 20 μL volumes comprised of 10 μL PrimeSTAR Max DNA Polymerase (TaKaRa, Tokyo, Japan), 1 μL of each primer (10 mmol/L), 1 μL of template DNA solution (70 ng/μL), and 7 μL double distilled water. Amplifications were conducted as follows: 34 cycles of denaturation at 94 °C for 30 s, annealing at 59 °C for 30 s, and extension at 72 °C for 1 min. All the PCR products were checked by electrophoresis on a 1.2% agarose gel and bidirectional sequencing was completed by TSINGKE (Beijing, China).

### ISSR PCR amplification

A total of sixteen primers from the University of British Columbia Biotechnology Laboratory Primer kit No.9 were tested for PCR and nine (Additional file 1: Table S2) that could produce reproducible, clear, polymorphic electrophoretic bands were chosen for further analysis. PCR was performed in 20 μL volumes comprised of 10 μL Premix Taq™ (Ex Taq™ Version 2.0 plus dye) (TaKaRa, Tokyo, Japan), 2 μL of primer (10 mmol/L), 1 μL of template DNA solution (70 ng/μL), and 7 μL double distilled water. Amplifications were carried out as follows: an initial denaturing at 94 °C for 3 min, followed by 34 cycles of denaturing at 94 °C for 30 s, annealing at an optimized temperature for 30 s, and extension at 72 °C for 1 min, with a final extension 7 min at 72 °C. All PCR products were electrophoretically separated on 2% agarose.

### Data analysis

#### *COI* gene data analysis

*COI* sequences were edited manually with BioEdit v.7.0.9 to produce consensus sequences of 685 bp for each specimen [37]. All indices for sequence polymorphic sites, DNA polymorphism, genetic differentiation, neutrality tests [Tajima’s *D* [38] and Fu’s *Fs* [39] ], and haplotype analyses were executed using DnaSp v.5.10 [40]. A haplotype network, which included haplotype frequencies, was calculated using Network v.4.6 [41]. Intra- and inter-specific genetic distances and transition/transversion ratios in each codon were computed based on *COI* gene sequences using MEGA v.7.0 [42]. A population phylogenetic tree based on genetic distances was constructed using the Neighbor-Joining tree model in MEGA v.7.0. Analysis of molecular variance (AMOVA) was performed with Arlequin software v.3.5.2 [43].

#### ISSR data analysis

Amplified ISSR fragments were scored as present (1) or absent (0) according to the molecular weight (bp) and the resulting matrix of binary values was used for further analyses. The observed number of alleles (*Na*), effective number of alleles (*Ne*), Nei’s gene diversity (*H*), Shannon’s information index (*I*), the percentage of polymorphic bands (PPB), total gene diversity (*Ht*), genetic diversity within populations (*Hs*), coefficient of gene differentiation (*Gst*), and Gene flow (*Nm*) were calculated using POPOGENE v.1.31 [44]. Cluster analysis was used to construct dendrograms using the UPGMA (unweighted pair-group method with arithmetic averages) in NTSYSpc v.2.1 [45]. To estimate the existence of isolation among 20 *C. oryzae* populations, the geographic distances between them were calculated using the google-maps-distance-calculator (www. daftlogic.com/projects-google-maps-distance-calculator. htm) (Additional file 1: Table S3). The correlation between geographic and genetic distances was further analyzed using the MXCOMP program and a Mantel test in NTSYSpc v.2.1.

## Supplementary information

**Additional file 1.** Additional tables.

## Data Availability

The authors declare that the data supporting the finding of this study are available in the article and its additional files.

## Abbreviations

mtDNA: Mitochondrial DNA
ISSR: Inter-simple sequence repeat
gDNA: Genomic DNA
*COI*: Mitochondrial gene Cytochrome Oxidase I
AMOVA: Analysis of molecular variance
*Na*: The observed number of alleles
*Ne*: Effective number of alleles
*H*: Nei’s gene diversity
*I*: Shannon’s information index
PPB: The percentage of polymorphic bands
*Ht*: Total gene diversity
*Hs*: Genetic diversity within populations
*Gst*: Coefficient of gene differentiation
*Nm*: Gene flow
UPGMA: Unweighted pair-group method with arithmetic averages
*Hd*: Haplotype diversity
*k*: The average number of differences
*Π*: Nucleotide diversity
