# Supplementary material for: Genetic diversity and differentiation of populations of Chlorops oryzae (Diptera, Chloropidae)
Source: BMC Ecol. 2020 Apr 15;20:22. doi: 10.1186/s12898-020-00293-8 (PMC7160969; doi:10.1186/s12898-020-00293-8)
Supplement: Supplementary file 1 — Additional file 1. Additional tables. [file 12898_2020_293_MOESM1_ESM.docx]

Table S1. Information on the *C. oryzae* samples used in this study.

| Populations | Collection sites | Collection sites | Location coordinates | Altitude (m) | Collection dates | Number of specimens |
| --- | --- | --- | --- | --- | --- | --- |
| TY | TY1 | Chengguishan Village, Sanyanggang Town, Taoyuan County | E111.3554 N28.9734 | 70 | 07/08/2018 | 8 |
|  | TY2 | Laoguanping Village, Wuxi River, Qinglin Township, Taoyuan County | E111.3840 N29.0280 | 53 | 07/08/2018 | 8 |
|  | TY3 | Wumazhai Village, Niwotan Township, Taoyuan County | E111.3409 N28.8712 | 78 | 05/08/2018 | 8 |
| ZZ | ZZ1 | Huatian Village, Longtan Town, Zhuzhou County | E113.1388 N27.3454 | 97 | 15/08/2018 | 8 |
|  | ZZ2 | Xichong Village, Longtan Town, Zhuzhou County | E113.1397 N27.3787 | 117 | 15/08/2018 | 8 |
|  | ZZ3 | Huashi Village, Gantian Town, Zhuzhou County | E113.0671 N27.4496 | 82 | 14/08/2018 | 8 |
| LS | LS1 | Fuxing Village, Maoping Township, Longshan County | E109.3122 N29.1842 | 874 | 09/08/2018 | 8 |
|  | LS2 | Yanpomen Village, Zhaoshi Town, Longshan County | E109.2902 N29.1742 | 663 | 09/08/2018 | 8 |
|  | LS3 | Zhuke Village, Maoping Township, Longshan County | E109.3027 N29.1957 | 874 | 09/08/2018 | 8 |
| YS | YS1 | Lilie Village, Shouche Town, Yongshun County | E109.7593 N29.1152 | 387 | 25/07/2018 | 8 |
|  | YS2 | Xinyin Village, Songbai Town, Yongshun County | E110.0787 N28.9158 | 721 | 25/07/2018 | 8 |
|  | YS3 | Deciduous Cave Village, Shidi Town, Yongshun County | E110.1066 N29.0371 | 486 | 24/07/2018 | 8 |
| XT | XT1 | Heping Village, Huashi Town, Xiangtan County | E112.4259 N27.3152 | 68 | 08/08/2018 | 8 |
|  | XT2 | Qingfeng Village, Yunhuqiao Town, Xiangtan County | E112.6686 N27.8630 | 89 | 07/08/2018 | 8 |
|  | XT3 | Heyeba Village, Tanjiashan Town, Xiangtan County | E112.5504 N27.3910 | 146 | 09/08/2018 | 8 |
| HS | HS1 | Hanshou County | E111.9704 N28.9055 | 34 | 30/07/2018 | 8 |
|  | HS2 | Baizhushan Village, Fengjiapu Town, Hanshou County | E111.8539 N28.6712 | 69 | 31/07/2018 | 8 |
|  | HS3 | Wanshou Village, Junshanpu Town, Hanshou County | E112.1507 N28.7080 | 101 | 30/07/2018 | 8 |
| HD | HD1 | Fenghuang Village, Yangqiao Town, Hengdong County | E113.0929 N27.1319 | 103 | 25/07/2018 | 8 |
|  | HD2 | Shimen Village, Yangqiao Town, Hengdong County | E113.0816 N27.1417 | 227 | 25/07/2018 | 8 |
| JS | JS1 | Paishan Village, Taiping Town, Jishou City | E109.9153 N28.3525 | 230 | 09/08/2018 | 8 |
|  | JS2 | Unity Village, Majingao Town, Jishou City | E109.8373 N28.4516 | 355 | 09/08/2018 | 8 |
| SM | SM1 | Zengjiaya Village, Taiping Town, Shimen County | E111.1040 N29.8434 | 370 | 16/08/2018 | 8 |
|  | SM2 | Chongfuqiao Village, Yanchi Township, Shimen County | E111.0321 N29.8517 | 220 | 15/06/2019 | 8 |
|  | SM3 | Xiantai Village, Taiping Town, Shimen County | E111.0908 N29.9259 | 270 | 14/06/2019 | 8 |
| DC | DC1 | Xiangjiaxiang Village, Xiejiapu Town, Dingcheng District, Changde City | E111.7850 N28.8367 | 40 | 03/08/2018 | 8 |
|  | DC2 | Shijiabei Village, Xiejiapu Town, Dingcheng District, Changde City | E111.4939 N28.5003 | 33 | 23/05/2019 | 8 |
|  | DC3 | Dingcheng District, Changde City | E111.6515 N28.9215 | 37 | 28/05/2019 | 8 |
| XX | XX1 | Shizichong Village, Meiqiao Town, Xiangxiang City | E112.5890 N27.6829 | 73 | 27/08/2018 | 8 |
|  | XX2 | Shuangjiang Village, Lishan Town, Xiangxiang City | E112.3985 N27.6570 | 110 | 28/08/2018 | 8 |
|  | XX3 | Xinan Village, Dongshan Township, Xiangxiang City | E112.5510 N27.7440 | 43 | 28/05/2019 | 8 |
| YL | YL1 | Dongshuixi Village, Mingxikou Town, Yuanling County | E110.3538 N28.6538 | 271 | 24/08/2018 | 8 |
| YX | YX1 | Wangling Town, Youxian County | E113.1509 N27.1619 | 231 | 11/09/2018 | 8 |
|  | YX2 | Beiping Village, Wangling Town, Youxian County | E113.2525 N27.1439 | 118 | 28/08/2018 | 8 |
|  | YX3 | Youxian County | E113.3457 N27.0002 | 102 | 30/05/2019 | 8 |
| SS | SS1 | Nan Village, Yintian Town, Shaoshan City | E112.6109 N27.8791 | 56 | 03/09/2018 | 8 |
|  | SS2 | Tuantian Village, Yanglin Township, Shaoshan City | E112.4552 N27.9588 | 123 | 04/09/2018 | 8 |
|  | SS3 | Shaoyang Village, Shaoshan Township, Shaoshan City | E112.4680 N27.8635 | 152 | 04/09/2018 | 8 |
| LL | LL1 | Tiehekou Village, Chashan Town, Liling City | E113.3921 N27.6414 | 59 | 11/09/2018 | 8 |
|  | LL2 | Mingyue Town, Liling City | E113.4248 N27.4663 | 89 | 05/09/2018 | 8 |
|  | LL3 | Sifen Town, Liling City | E113.5031 N27.5202 | 68 | 28/05/2019 | 8 |
| TJ | TJ1 | Jiujiaduan Village, Shiniujiang Town, Taojiang County | E112.1701 N28.4248 | 68.4 | 17/05/2019 | 8 |
|  | TJ2 | Niutanhe Village, Taohuajiang Town, Taojiang County | E112.1654 N28.5638 | 64.3 | 17/05/2019 | 8 |
|  | TJ3 | Zhangziqing Village, Lucidu Town, Taojiang County | E111.9787 N28.4501 | 89.7 | 17/05/2019 | 8 |
| LH | LH1 | Shangsheng Village, Qijiang Town, Longhui County | E111.0057 N27.4585 | 400 | 31/05/2019 | 8 |
|  | LH2 | Daoqun Village, Jinshiqiao Town, Longhui County | E110.9242 N27.5857 | 420 | 31/05/2019 | 8 |
|  | LH3 | Wuluo Village, Qijiang Town, Longhui County | E110.5446 N27.3459 | 474 | 31/05/2019 | 8 |
| GZ |  | Chenliang Village, Huaxi District, Guiyang City, Guizhou Province | E106.3910 N26.2712 | 1096 | 09/06/2019 | 8 |
| ZJ |  | Jingning Shezu Autonomous County, Lishui City, Zhejiang Province | E119.6358 N27.9732 | 300-900 | 05/06/2019 | 24 |
| NX | NX1 | Shuangjiangkou Town, Ningxiang City | E112.6319 N28.0044 | 36 | 28/05/2019 | 8 |
|  | NX2 | Lijingpu Township, Ningxiang City | E112.5883 N28.2350 | 89 | 29/05/2019 | 8 |
|  | NX3 | Huilongpu Town, Ningxiang City | E112.4587 N28.2131 | 69 | 28/05/2019 | 8 |

Table S2. Sequence of ISSR primers used to amplify *C. oryzae* DNA.

| primer | sequence (5'-3') | annealing temperature (℃) |
| --- | --- | --- |
| ISSR1 | CACACACACACAAC | 42 |
| ISSR2 | CACACACACACAAG | 42 |
| ISSR3 | CACACACACACAGG | 43.8 |
| ISSR4 | AGAGAGAGAGAGAGAGTA | 50.2 |
| ISSR5 | AGAGAGAGAGAGAGAGTC | 50.2 |
| ISSR6 | GAGAGAGAGAGAGG | 43.8 |
| ISSR7 | CTCCTCCTCCTCGC | 46.7 |
| ISSR8 | AGAGAGAGAGAGAGAGAT | 50.2 |
| ISSR9 | CTCTCTCTCTCTCTCTAC | 50.2 |

Table S3. Geographical distance (km) among *C. oryzae* populations.

|  | TY | ZZ | LS | YS | XT | HS | HD | JS | SM | DC | XX | YL | YX | SS | LL | TJ | LH | GZ | ZJ | NX |
| --- | --- | --- | --- | --- | --- | --- | --- | --- | --- | --- | --- | --- | --- | --- | --- | --- | --- | --- | --- | --- |
| TY |  |  |  |  |  |  |  |  |  |  |  |  |  |  |  |  |  |  |  |  |
| ZZ | 245.07 |  |  |  |  |  |  |  |  |  |  |  |  |  |  |  |  |  |  |  |
| LS | 201.86 | 423.75 |  |  |  |  |  |  |  |  |  |  |  |  |  |  |  |  |  |  |
| YS | 134.42 | 357.01 | 68.53 |  |  |  |  |  |  |  |  |  |  |  |  |  |  |  |  |  |
| XT | 197.67 | 57.87 | 367.87 | 301.96 |  |  |  |  |  |  |  |  |  |  |  |  |  |  |  |  |
| HS | 65.33 | 188.26 | 266.13 | 198.11 | 148.30 |  |  |  |  |  |  |  |  |  |  |  |  |  |  |  |
| HD | 264.33 | 28.45 | 435.82 | 370.31 | 68.51 | 210.51 |  |  |  |  |  |  |  |  |  |  |  |  |  |  |
| JS | 157.54 | 337.90 | 103.58 | 69.84 | 280.31 | 210.65 | 346.20 |  |  |  |  |  |  |  |  |  |  |  |  |  |
| SM | 105.65 | 340.67 | 188.13 | 142.24 | 298.55 | 152.43 | 362.66 | 201.07 |  |  |  |  |  |  |  |  |  |  |  |  |
| DC | 35.82 | 209.43 | 233.05 | 164.80 | 163.16 | 34.02 | 229.14 | 177.14 | 136.41 |  |  |  |  |  |  |  |  |  |  |  |
| XX | 180.34 | 68.36 | 355.35 | 288.72 | 19.40 | 129.29 | 84.11 | 270.75 | 280.15 | 145.41 |  |  |  |  |  |  |  |  |  |  |
| YL | 103.81 | 305.56 | 118.30 | 54.84 | 249.54 | 160.39 | 317.51 | 54.49 | 152.84 | 126.45 | 237.24 |  |  |  |  |  |  |  |  |  |
| YX | 277.73 | 34.84 | 451.50 | 385.73 | 83.75 | 222.32 | 16.58 | 362.47 | 374.71 | 242.29 | 98.25 | 333.20 |  |  |  |  |  |  |  |  |
| SS | 162.94 | 82.20 | 344.84 | 277.35 | 42.17 | 108.53 | 102.26 | 264.61 | 260.41 | 127.38 | 22.92 | 227.51 | 114.96 |  |  |  |  |  |  |  |
| LL | 257.69 | 36.26 | 444.60 | 376.96 | 88.08 | 196.49 | 57.09 | 363.20 | 347.26 | 221.86 | 92.98 | 327.30 | 52.52 | 99.80 |  |  |  |  |  |  |
| TJ | 90.09 | 156.69 | 284.35 | 215.89 | 115.04 | 33.27 | 178.04 | 218.32 | 184.60 | 54.33 | 96.15 | 172.25 | 190.36 | 75.79 | 167.63 |  |  |  |  |  |
| LH | 174.41 | 226.42 | 242.85 | 192.29 | 170.29 | 184.43 | 226.70 | 140.09 | 269.43 | 164.53 | 168.55 | 140.35 | 243.28 | 173.19 | 258.36 | 169.20 |  |  |  |  |
| GZ | 574.05 | 679.35 | 432.92 | 468.06 | 626.84 | 618.45 | 672.73 | 418.32 | 610.26 | 587.51 | 627.55 | 472.77 | 688.34 | 632.99 | 713.78 | 615.91 | 459.81 |  |  |  |
| ZJ | 817.07 | 645.97 | 1018.79 | 951.44 | 699.98 | 753.70 | 652.89 | 958.51 | 860.17 | 787.47 | 701.74 | 912.53 | 637.65 | 700.63 | 612.14 | 740.64 | 869.90 | 1325.16 |  |  |
| NX | 147.72 | 100.71 | 338.33 | 270.08 | 69.88 | 87.80 | 124.28 | 264.56 | 240.08 | 111.90 | 50.99 | 223.10 | 135.15 | 28.27 | 109.97 | 57.66 | 187.15 | 645.42 | 695.28 |  |
